# Tuberculosis: The Master Masquerader

**DOI:** 10.7759/cureus.80221

**Published:** 2025-03-07

**Authors:** Abarna Poornima, Adarsha Shetty, Nandakishore Baikunje, Giridhar Belur, Chandramouli M T

**Affiliations:** 1 Pulmonary Medicine, K. S. Hegde Medical Academy (KSHEMA), Mangaluru, IND

**Keywords:** ebus and interventional bronchoscopy, left sided pleural effusion, medical thoracoscopy, pleural tuberculosis, risk of malignancy

## Abstract

A 54-year-old male presented with a large, left-sided hemorrhagic exudative pleural effusion characterized by lymphocytic predominance and a low adenosine deaminase (ADA) level. A pleural biopsy was performed via medical thoracoscopy. The results of histopathology and GeneXpert-MTB/RIF (*Mycobacterium tuberculosis*/Rifampicin) (Cepheid, Sunnyvale, California, USA) were inconclusive. PET-CT imaging revealed heightened metabolic activity in the mediastinal lymph nodes, further supported by endobronchial ultrasound (EBUS) and transbronchial needle aspiration (TBNA), showing granulomatous lymphadenitis with caseous necrosis. The patient was diagnosed with tuberculous pleural effusion and showed clinical improvement after initiating anti-tubercular therapy.

## Introduction

Tuberculous pleural effusion (TPE) is the second most common extrapulmonary manifestation of tuberculosis (TB). The incidence varies between regions, ranging from 3% to 30%. It is 3-5% in non-endemic areas, while in endemic countries like India, it is 29.7%. The incidence is also higher among individuals with HIV infection [1,2]. TPE results from delayed hypersensitivity to mycobacterial antigens in the pleural space or the rupture of a subpleural focus of pulmonary disease into the pleural cavity [3,4]. An acute process may cause a neutrophilic exudative pleural effusion, while a lymphocytic exudative effusion prompts consideration of TB and malignancy. In normal individuals, lymphocytes comprise 20% of the cell count in pleural fluid, while macrophages account for 75%. Mesothelial cells, neutrophils, and eosinophils together constitute the remaining 5%. A combined pleural fluid lymphocyte-to-neutrophil ratio greater than 0.75, along with elevated pleural fluid adenosine deaminase (ADA) levels (≥40 IU), has demonstrated a sensitivity of 88% and specificity of 95% in diagnosing TPE [5].

Other diagnostic modalities for the diagnosis of TPE include nucleic acid amplification for *Mycobacterium tuberculosis (MTB*), which has a sensitivity of 49.5% and a specificity of 98.9%; histopathological evaluation of pleural biopsy for granulomas, which has a sensitivity of 72-80%; and MTB culture from the biopsy sample, which has a sensitivity of 60-67% [6]. Although TPE can resolve spontaneously, about half of the untreated cases may subsequently develop active TB [7]. A lymphocytic effusion with a low ADA level (<40 IU) can be seen in malignancy; therefore, malignancy should be ruled out before considering TB in cases of large hemorrhagic pleural effusions [8]. This case report underscores an unusual presentation of a large, hemorrhagic pleural effusion with low ADA levels in TB.

## Case presentation

**Figure 1:**
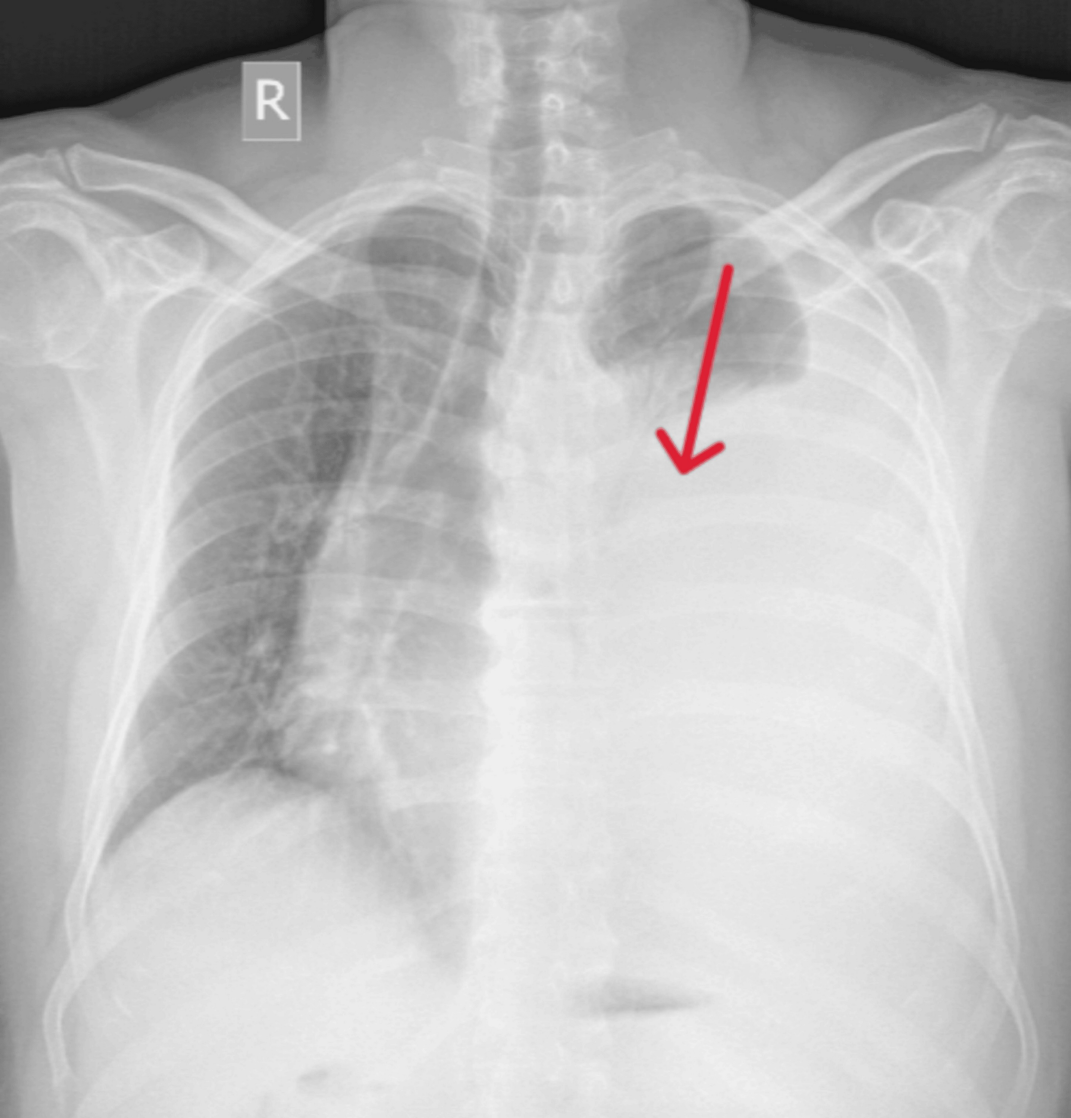
Chest X-ray showing a large left-sided pleural effusion (red arrow).

A 54-year-old male presented with breathlessness on exertion for two weeks. It was not associated with chest pain, fever, or cough. On physical examination, vitals were stable. The respiratory system examination revealed tracheal deviation to the right side, asymmetrical chest expansion, reduced tactile vocal fremitus, a dull note on percussion, and an absence of breath sounds over the left middle and basal lung fields, suggesting a left-sided pleural effusion. A chest X-ray from an outside hospital showed a large left-sided pleural effusion, prompting further evaluation (Figure 1).

Routine blood investigations were normal, except for the raised ESR. Diagnostic thoracentesis showed hemorrhagic, exudative, lymphocytic effusion with a low ADA level. Pleural fluid acid-fast bacilli (AFB) staining, GeneXpert- Mycobacterium tuberculosis/Rifampicin (MTB/RIF) (Cepheid, Sunnyvale, California, USA), and bacterial culture were inconclusive (Table 1).

**Table 1:** Pleural fluid analysis. HPF: High power field; TLC: Total leucocyte count; DLC: Differential leucocyte count; MTB: Mycobacterium tuberculosis; AFB: Acid-fast bacilli.

Pleural fluid parameters	Results	Reference range
Appearance	Reddish	-
Protein	4.5 g/dl	1-2 g/dl
Sugar	82 mg/dl	< 140 mg/dl
Lactate dehydrogenase	824 U/L	-
ADA	6.43 U/L	< 30 U/L
Cytology	RBC: Plenty/HPF TLC- 830 cells/cumm DLC: Lymphocytes 74%, Neutrophils 20%, and Reactive mesothelial cells 06%	-
Acid Fast Bacilli	No AFB seen	-
GeneXpert MTB/RIF	MTB not detected	-
Bacterial culture	No growth	-

**Figure 2:**
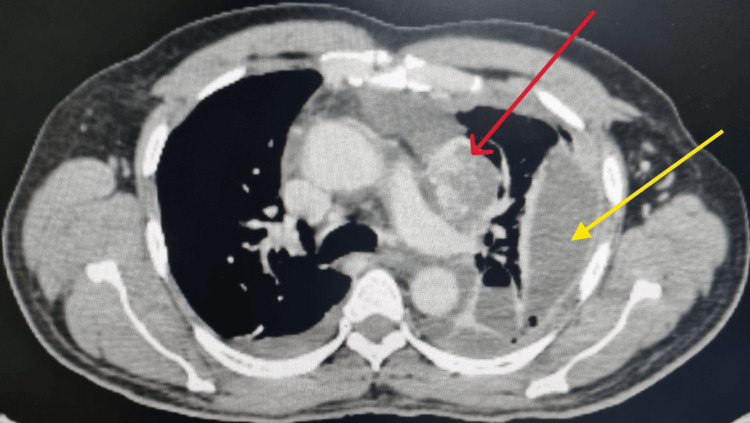
Thoracic computed tomography showing a necrotic mass lesion adjacent to the left main pulmonary artery (red arrow) and pleural effusion (yellow arrow).

A CT scan of the thorax revealed a superior mediastinal necrotic mass lesion adjacent to the left main pulmonary artery, paratracheal and subcarinal lymph nodes, and pleural effusion (Figure 2).

**Figure 3:**
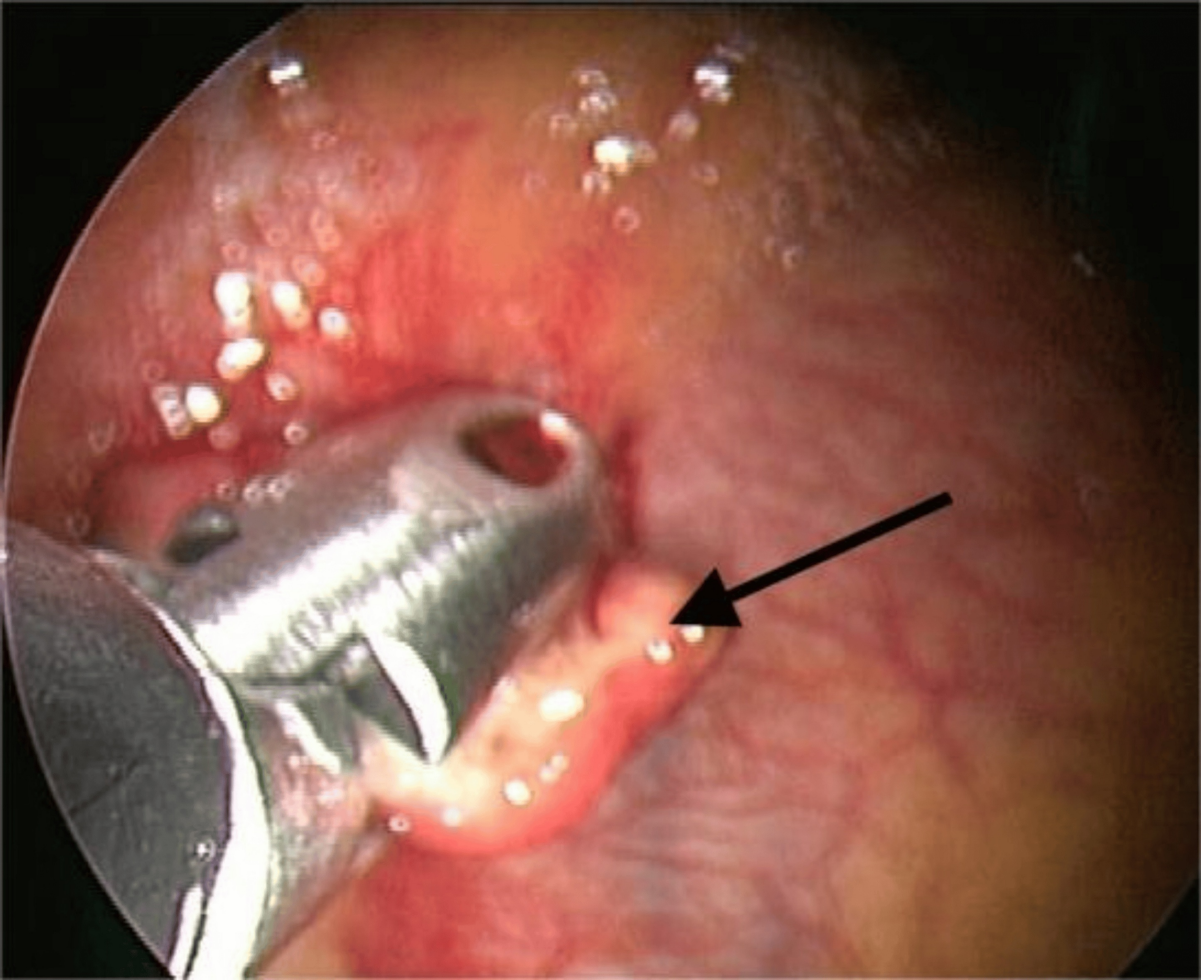
Medical thoracoscopy image showing a macronodule in the costal pleura (black arrow).

Bronchoscopy with bronchoalveolar lavage for AFB staining and GeneXpert-MTB/RIF was inconclusive. Medical thoracoscopy showed erythematous mucosa and a macronodule in the costal pleura (Figure 3).

A biopsy was taken, an intercostal drain (ICD) was inserted, and histopathology revealed chronic inflammation. Both AFB staining and GeneXpert for MTB were inconclusive. However, the patient continued to have recurrent hemorrhagic pleural effusion.

**Figure 4:**
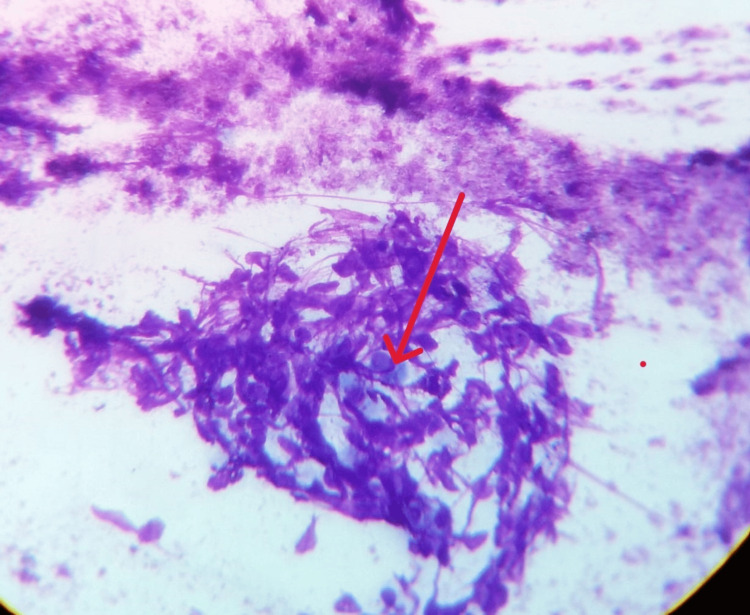
Methylene blue stain, 40x power cellular smear of subcarinal lymph node showing granuloma with caseous necrosis (red arrow).

Due to suspicion of malignancy, PET-CT was performed, revealing increased uptake in bilateral paratracheal, paraaortic, and subcarinal nodes. Endobronchial ultrasound-transbronchial needle aspiration (EBUS-TBNA) from the subcarinal lymph node was performed. The TBNA sample showed a negative GeneXpert result, but the cytological examination revealed granulomatous lymphadenitis with caseous necrosis (Figure 4).

**Figure 5:**
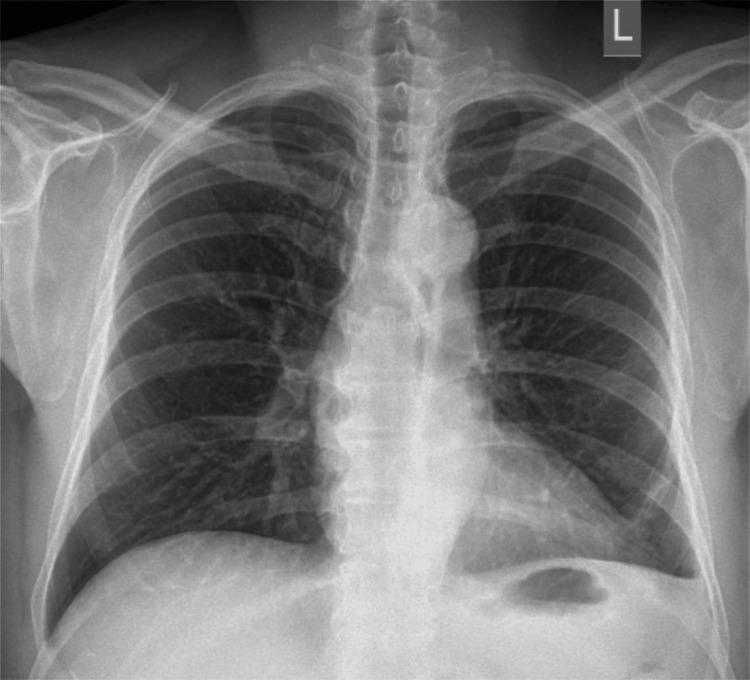
Chest X-ray after four weeks of ATT showing complete resolution of the effusion. ATT: Antitubercular Treatment.

The patient was started on antitubercular treatment (ATT) after ruling out malignancy according to National Tuberculosis Elimination Program (NTEP) guidelines [9]. The ICD was removed once consecutive drains were reduced. The patient’s symptoms improved, and he was discharged. A chest X-ray during consecutive follow-ups revealed complete resolution of the pleural effusion (Figure 5).

## Discussion

TPE is the most frequent cause of pleural effusion in TB-endemic areas. In the absence of pulmonary TB, TPE is diagnosed by diagnostic thoracentesis and pleural fluid analysis. Hemorrhagic, large pleural effusions are frequently associated with malignancies. Globally, TB hemorrhagic pleural effusion is reported in 3 to 5% of cases [10]. This case report underscores the critical importance of considering TB as a possible diagnosis when a patient presents with a hemorrhagic, low ADA, and exudative pleural effusion, especially after malignancy has been ruled out. This consideration is particularly relevant in countries where TB is endemic.

TPE is usually straw-colored, unilateral, and of small to moderate volume. However, in rare cases, it can present as a large, hemorrhagic pleural effusion. To date, only six cases of hemorrhagic TPE have been reported, with only one case involving a massive hemorrhagic pleural effusion. Malignancy is the primary consideration when assessing a massive hemorrhagic effusion, necessitating a thorough workup [10-12].

In regions with high TB prevalence, the presence of a lymphocyte-predominant exudate with elevated ADA levels has a positive predictive value of 98% for TB [13]. Conversely, in areas with low TB prevalence, the absence of elevated ADA levels carries a negative predictive value of 98% and effectively rules out suspected pleural TB [14]. However, low ADA levels in TB-endemic areas do not definitively exclude the diagnosis of TB. Chandra SC et al. conducted a study involving twenty-eight patients with exudative lymphocytic pleural effusion and low ADA levels. Through thoracoscopic pleural biopsy, malignancy was diagnosed in 60.7% of the patients, while TB was diagnosed in 28.6% [15]. Similarly, Kim SB et al. studied 192 patients with TPE, of whom 18.8% exhibited low ADA levels. In this cohort, low ADA activity was associated with advanced age and critical illness [16]. Their findings underscore that low ADA levels do not completely rule out TB, highlighting the need for further diagnostic workup, including medical thoracoscopy. The present case report highlights the diverse presentations of TB disease and the diagnostic challenges that should be considered in evaluating pleural effusion.

Early diagnosis and treatment of TB effusion are crucial because TB generally responds well to treatment when identified promptly. Therefore, despite the initial clinical resemblance to malignancy, considering TB in such cases can lead to timely intervention and better patient outcomes.

## Conclusions

Tuberculous pleuritis can present with unusual characteristics, such as a large hemorrhagic pleural effusion, which requires a high index of clinical suspicion. A combination of diagnostic techniques, including imaging studies, molecular diagnostics, medical thoracoscopy, and EBUS-TBNA, is essential for arriving at an accurate diagnosis and ruling out malignancy. In regions where TB is endemic, it is critical to consider TB in cases of hemorrhagic pleural effusion with low ADA levels. Prompt initiation of anti-tubercular therapy and close monitoring of clinical progress are key to effectively managing TPE. Ongoing follow-up is necessary to detect any recurrence and ensure full recovery from the disease.
